# Pneumococcal sepsis presenting as acute compartment syndrome of the lower limbs: a case report

**DOI:** 10.1186/1752-1947-3-55

**Published:** 2009-02-09

**Authors:** Sudeendra Doddi, Tarun Singhal, Prakash Sinha

**Affiliations:** 1 Department of General Surgery, Princess Royal University Hospital, Farnborough Common, Orpington, Greater London, BR6 8ND, UK

## Abstract

**Introduction:**

Acute compartment syndrome is a surgical emergency requiring immediate fasciotomy. Spontaneous onset of acute compartment syndrome of the lower limbs is rare. We present a very rare case of pneumococcal sepsis leading to spontaneous acute compartment syndrome.

**Case presentation:**

A 40-year-old Caucasian man presented as an emergency with spontaneous onset of pain in both legs and signs of compartment syndrome. This was confirmed on fasciotomy. Blood culture grew *Streptococcus pneumoniae*.

**Conclusion:**

Sepsis should be strongly suspected in bilateral acute compartment syndrome of spontaneous onset.

## Introduction

Acute compartment syndrome of the limbs, if diagnosed late or left untreated, can have grave consequences such as myonecrosis, contractures, functional impairment, limb amputation, renal failure and death. Hence, prompt decompression by way of fasciotomy is vital. Diagnosis of compartment syndrome is essentially clinical-pain out of proportion to the clinical situation, weakness, pain on passive stretch of the muscles, hypoaesthesia and tenseness of the compartment [1]. The cause of the compression syndrome is addressed once the pressure is released. There have been a few case reports of *Streptococcus pyogenes *causing acute spontaneous compartment syndrome [2]. However, this is the first report of spontaneous acute bilateral lower leg compartment syndrome caused by sepsis due to *Streptococcus pneumoniae*.

## Case presentation

A 40-year-old previously well Caucasian man presented as an emergency with a 1-day history of vomiting and pain in both legs. There was no history of trauma or infection in the lower limbs and he was not on any regular medication. He did admit to having a sore throat for the past week for which he did not seek medical attention.

On examination, he was apyrexial and normotensive with a heart rate of 120/minute. His tonsils were enlarged though not inflamed. There were no meningeal signs or skin rash. Chest and abdominal examination were normal. Both his legs were swollen, tense and tender. The dorsalis pedis pulse was palpable equally. There was no paraesthesia or weakness in his legs. He weighed 70 kg.

There were several abnormalities in his blood tests (Table 1). The significant abnormalities noted were: white blood cell count (WBC) 29.4 × 10^9^/L (normal range, 4.0 to 11.0), neutrophils 25.4 × 10^9^/L (2.0 to 7.5), haemoglobin 21.9 g/dL (13.0 to 18.0), urea 12.1 mmol/L (2.1 to 7.1), creatinine 219 μmol/L (84 to 114).

**Table 1:** Results of blood investigations at the time of admission

Parameter	Level	Normal range
Sodium	140 mmol/L	136 to 145
Potassium	5.1 mmol/L	3.5 to 5.1
Urea	12.1 mmol/L	2.1 to 7.1
Creatinine	219 μmol/L	84 to 114
Total protein	50 g/L	64 to 83
Albumin	25 g/L	34 to 48
Alkaline phosphatase	36 U/L	25 to 114
Gamma GT	13 U/L	7 to 59
Aspartate transferase	24 U/L	22 to 59
Bilirubin	6 μmol/L	5 to 21
Amylase	16 U/L	20 to 104
C-reactive protein	10 mg/L	0 to 10
WBC	29.4 × 10^9^/L	4.0 to 11.0
RBC	7.46 × 10^12^/L	4.5 to 6.5
HB	21.9 g/dL	13.0 to 18.0
HCT	0.645 L/L	0.400 to 0.520
MCV	86.4 fL	80 to 100
MCH	29.4 pg	27.0 to 32.0
Platelets	286 × 10^9^/L	150 to 450
Neutrophils	25.4 × 10^9^/L	2.0 to 7.5
Eosinophils	0.0 × 10^9^/L	0.04 to 0.4
Basophils	0.2 × 10^9^/L	0.0 to 0.1
Monocytes	1.2 × 10^9^/L	0.2 to 0.8
Lymphocytes	2.5 × 10^9^/L	1.5 to 4.0
Lactate	5.52 mmol/L	0.50 to 2.22

Gamma GT, gamma glutamyl transferase; HB, haemoglobin; HCT, haematocrit; MCV, mean corpuscular volume; MCH, mean corpuscular haemoglobin; RBC, red blood cell count; WBC, white blood cell count

The platelet count, electrolytes, liver function test and clotting were normal. Blood, urine and throat swabs were taken for microbiology. The urine dipstick, chest X-ray and electrocardiogram (ECG) were normal. Arterial blood gas revealed compensated metabolic acidosis.

It was observed that the analgesic requirement for the pain in his legs was escalating and the leg swelling was progressive. The patient developed pain on passive stretch, decreased saturation on pulse oximetry of both toes and increasing firmness in the legs. Clinically, acute compartment syndrome was suspected and fasciotomy of both the legs was performed using the double incision technique to decompress all four compartments. Herniation of the muscles on skin incision confirmed raised compartment pressure. The muscles in both the legs were viable. By now, he was oliguric and hypotensive. Central venous pressure monitoring revealed he was adequately filled. He was thought to be septic and noradrenaline was commenced at 14 mcg per minute in the intensive care unit (0.2 mcg per kg per minute) for inotropic support. He was commenced on benzylpenicillin 1.2 g three times a day, clindamycin 600 mg three times a day and gentamicin 350 mg per day after seeking advice from the microbiologist.

Next morning, the noradrenaline was tailed off and his renal function improved. His blood cultures grew *S. pneumoniae *within 24 hours and they were found to be sensitive to penicillin. The wound swabs were negative. The fasciotomy wounds were closed 5 days later and he made an uneventful recovery.

## Discussion

Acute compartment syndrome is elevation of interstitial pressure beyond the vascular perfusion pressure in a closed fascial compartment that results in microvascular compromise and leads to muscle and nerve ischaemia and necrosis [2]. Common causes of acute compartment syndrome of the lower limbs are: tibial fractures, haemorrhage, reperfusion after vascular obstruction, vascular puncture in anticoagulated patients, vigorous exertion, lithotomy position and prolonged limb compression.

Normal resting intramuscular pressure is 0 to 8 mmHg. Pain and paraesthesia appear when the intracompartmental pressure (ICP) is about 20 to 30 mmHg. At an ICP of 30 mmHg, irreversible changes occur in 6 to 8 hours [3]. There are various techniques for direct percutaneous monitoring of ICP, but criteria have varied regarding the accepted useful diagnostic readings [4]. Whitesides *et al. *suggest that the perfusion of the compartment depends on the difference between the diastolic blood pressure and the ICP [5]. They recommend fasciotomy when this pressure difference, known as Delta p, is less than 30 mmHg. Matsen *et al. *demonstrated that the concept of a critical value above which decompression should be performed is of limited value [1]. Intracompartmental pressure measurement may have a role in the diagnosis of this condition in unconscious patients or those unable to co-operate [6]. Measurement of compartmental pressures, even if available, should not delay treatment. Diagnosis of ACS is essentially clinical.

An open fasciotomy using the double incision technique is performed to decompress the four compartments in the leg-anterior, lateral, superficial and deep posterior. This technique has the advantage in that it is quicker and does not damage the neurovascular structures [7]. It is important to make an adequate length of incision for effective decompression. Close monitoring of the wound is needed as further debridement of necrotic tissue may be required. The wound may be closed by skin closure (secondary, delayed primary or primary), skin grafting or flap coverage. If early secondary closure is contemplated, intracompartmental pressure monitoring may be required.

Bacterial infection causing acute compartment syndrome has been reported. There have been a few case reports of group A streptococcus causing acute compartment syndrome [8]. *S. pneumoniae *causes a broad spectrum of diseases: upper and lower respiratory tract infections, otitis media, sinusitis, meningitis, spontaneous bacterial peritonitis and post-splenectomy sepsis. The presence of a capsule allows it to escape phagocytosis, resulting in an intense inflammatory response in hosts who are immunologically naïve. Colonisation of the oropharynx by bacterial adherence to human pharyngeal cells is usually the first step. Penicillin remains the drug of choice for strains that are fully sensitive or have moderately decreased susceptibility to penicillin whereas cefotaxime and ceftriaxone are the first line alternatives in cases with higher levels of resistance. Blood culture is the most important tool for establishing a definitive diagnosis [9].

The mechanism of acute compartment syndrome in the setting of sepsis is unclear. Systemic capillary leak syndrome is a very rare condition characterised by increased systemic capillary leakage resulting in hypovolemic shock and compartment syndrome [10]. Sepsis could precipitate a similar situation: loss of integrity of the microcirculation, fluid exudation into the interstitial space, oedema formation, and muscle swelling and raised intracompartmental pressure. However, why this phenomenon is more pronounced in some compartments than others is unknown.

## Conclusion

Unexplained severe pain in the lower limbs should alert one to compartment syndrome even if there is no known aetiology. Early fasciotomy is essential to save the limb. One should consider sepsis early on, especially if there are signs of systemic inflammatory response, and institute broad-spectrum antibiotics and necessary supportive care to minimise morbidity and mortality.

## Abbreviations

ACS: acute compartment syndrome; ECG: electrocardiogram; gamma GT: gamma glutamyl transferase; HB: haemoglobin; HCT: haematocrit; Hg: mercury; ICP: intracompartmental pressure; MCV: mean corpuscular volume; MCH: mean corpuscular haemoglobin; RBC: red blood cell count; WBC: white blood cell count

## Consent

Written informed consent was obtained from the patient for publication of this case report and any accompanying images. A copy of the written consent is available for review by the Editor-in-Chief of this journal.

## Competing interests

The authors declare that they have no competing interests.

## Authors' contributions

SD was involved in patient care, collecting patient notes and the results of the investigations, literature search, and writing the manuscript. TS and PS performed the literature search and were major contributors in writing the manuscript. All authors were equally involved in conception and design of the paper; and have read and approved the final version of the manuscript for publication.
